# Non-invasive prenatal testing: a revolutionary journey in prenatal testing

**DOI:** 10.3389/fmed.2023.1265090

**Published:** 2023-11-09

**Authors:** Malak Abedalthagafi, Shahad Bawazeer, Romy I. Fawaz, A. Merrihew Heritage, Nouf M. Alajaji, Eissa Faqeih

**Affiliations:** ^1^Department of Pathology and Laboratory Medicine, Emory School of Medicine, Atlanta, GA, United States; ^2^King Salman Center for Disability Research, Riyadh, Saudi Arabia; ^3^Department of Medical Genetics, Children's Specialized Hospital, King Fahad Medical City, Riyadh, Saudi Arabia; ^4^Baylor Genetics, Houston, TX, United States; ^5^Department of Maternal Fetal Medicine, Women's Specialized Hospital, King Fahad Medical City, Riyadh, Saudi Arabia

**Keywords:** cffDNA, PCR, prenatal, pregnancy, inherited diseases, chromosomal disorders, NIPT, NIPS

## Abstract

Non-invasive prenatal testing (NIPT) is a pioneering technique that has consistently advanced the field of prenatal testing to detect genetic abnormalities and conditions with the aim of decreasing the incidence and prevalence of inherited conditions. NIPT remains a method of choice for common autosomal aneuploidies, mostly trisomy 21, and several monogenic disorders. The advancements in gene sequencing techniques have expanded the panel of conditions where NIPT could be offered. However, basic research on the impact of several genetic conditions lags behind the methods of detection of these sequence aberrations, and the impact of the expansion of NIPT should be carefully considered based on its utility. With interest from commercial diagnostics and a lack of regulatory oversight, there remains a need for careful validation of the predictive values of different tests offered. NIPT comes with many challenges, including ethical and economic issues. The scientific evidence, technical feasibility, and clinical benefit of NIPT need to be carefully investigated before new tests and developments are translated into clinical practice. Moreover, the implementation of panel expansion of NIPT should accompany expert genetic counseling pre- and post-testing.

## 1. Introduction

Non-invasive prenatal testing (NIPT) has revolutionized prenatal diagnostics with the aim of decreasing the incidence rates and prevalence levels of conditions that are inherited at birth. The term was first coined by Dr. Dennis Lo, who was the first to demonstrate the presence of cell-free fetal DNA (cffDNA) in maternal plasma and serum (1). Currently, NIPT remains a leading screening method for common viable autosomal aneuploidies, most of which are trisomy 21. In recent years, emerging technologies using genomic methods centered on next-generation sequencing have resulted in the expansion of prenatal analyses to the level that sub-chromosomal aneuploidies can also be detected, with NGS expansion enabling the detection of many single gene disorders. As the technology is still in its nascent phase, investigations on the validation and usefulness of varying methodologies used in already established NIPT tests are required to maximize its full potential.

NIPT has several advantages without the risks associated with invasive testing methodologies. These advancements have transformed the field of prenatal testing, and therefore, scientific societies across the globe have published recommendations on the ethical and justified application of the screening of cffDNA during pregnancy. In this review, we address the advancements in the field of NIPT and also touch upon the ethical questions and concerns that are an integral part of the discussion of advancements in technology (1).

## 2. Methodologies used in NIPT

This section discusses the state-of-the-art methodologies used for the determination of various abnormalities via NIPT. Table 1 summarizes the methods used in the analysis and detection of different gene/chromosomal abnormalities using NIPT.

**Table 1:** Methods used for the analysis of gene/chromosomal abnormalities using NIPT.

**Inheritance**	**Methodology used**	**Gene/chromosome**	**Disease/condition**
Autosomal dominant/*de novo* conditions	PCR-RED, dPCR, • ^**a**^Amplicon NGS • ^**a**^Bespoke testing for individual family	^a^*FGFR3* ^a^*FGFR2*	Achondroplasia (2) Thanatophoric dysplasia (3) Apert syndrome (4)
	PCR	*DMPK*	Myotonic dystrophy (5)
	Semi-qPCR; PCR and automated fragment analysis	*HTT*	Huntington (6)
Autosomal recessive conditions	^a^Amplicon NGS	a*CFTR*	Cystic fibrosis (7)
	dPCR	*PKHD1*	Autosomal recessive polycystic kidney disease (2)
	Polymorphic markers fluorescence PCR and fragment size analysis	*CYP21A2*	Congenital adrenal hyperplasia (8)
	qPCR	*HBB*	β-thalassemia (9)
	^a^Amplicon NGS	*HBB*	β-thalassemia (10)
	Population-based haplotyping-NIPT	*HBB*	*α and β*-thalassemia (11)
	ddPCR		*β-thalassemia mutations β+IVSI-110 and β039* (12)
	RMD—dPCR	*HBB*	β-thalassemia (13)
	dPCR	*HBB*	Sickle cell anemia (14)
	ddPCR	*MUT*	Methylmalonic acidemia (15)
	cSMART	*ATP7B*	Wilson disease (16)
	aNGS-RHDO	^a^ *CYP21A2*	Congenital adrenal hyperplasia (16)
	adPCR + NGS-RHDO	a*HBB*	β-thalassemia (17)
	^a^NGS-RHDO	^a^ *CFTR*	Cystic fibrosis (18)
	^a^NGS-RHDO	^a^ *SMN1 and SMN2*	Spinal muscular atrophy (18)
	cSMART	*MMACHC*	cblC type MMA (19)
Trisomies 21, 18, and 13	Microarray with DANSR assays	Chr 21, Chr 18, Chr13	Trisomy (20)
X-linked disorders	^a^NGS-RHDO	^a^ *DMD*	Duchene muscular dystrophy/Becker muscular dystrophy (18)
	dPCR	*F8, F9*	Hemophilia (18)

a Adapted from Jenkins et al. (18) and updated.

### 2.1. Massively parallel shotgun sequencing and chromosome selective sequencing

The techniques used to analyze complete genomic sequences are also applicable to the analysis of chromosomal abnormalities, copy-number variants (CNVs), and microdeletions used for NIPT (21). Most clinical trials have performed massively parallel shotgun sequencing (MPSS) and chromosome selective sequencing (CSS) (2). MPSS relies on the analysis of the complete genome and sequences of fetal and maternal cfDNA fragments, where fragments are quantified after being assigned to a chromosome. Therefore, a trisomic fetus will have a higher number of cfDNA fragments than the cfDNA expected in a euploid fetus (2).

Costs for MPSS are limited by restricting the sequencing to regions known to be involved in genetic abnormalities. These include regions on chromosomes 21, 18, 13, X, and Y. However, an analysis of 12 studies on MPSS and 6 on CSS by Yuval Yaron has shown that CSS has higher average failure rates of 3.56% vs. MPSS (22) with average failure rates of 1.58% (23).

SNP analysis differentiates between single nucleotide bases. SNP analysis through multiplex PCR can differentiate maternal DNA fragments from fetal fragments, which can be used to quantify the fetal fraction in CSS. SNP analysis has similar performance to MPSS and CSS but has a higher failure rate (24).

### 2.2. Microarray and digital PCR-based quantification

DNA microarray technology uses thousands of short nucleic acid sequences bound to a surface, which are used to quantify target nucleic acid sequences in a mixture via hybridization and subsequent detection of the hybridization events. As an alternative approach to CSS, microarray quantification has been proposed as a cost-effective and faster method that eliminates the risk of contamination, which is often observed in PCR. Additionally, it decreases the assay variability.

The digital PCR methodology is based on a single-molecule counting strategy to detect cfDNA. As digital PCR uses a single sample set, this procedure is not useful for large-scale analysis. Digital PCR has also been validated on T21 and is considered rapid and cost-effective in comparison to NGS. However, digital PCR requires adequate levels of cffDNA and would be useful after sufficient enrichment of the cffDNA (25). One of the limitations of digital PCR is that it cannot be used to detect low-grade mosaicism or other structural abnormalities in the chromosomes.

Recent data demonstrate the feasibility of digital PCR in analyzing DNA duplications and micro-deletions at resolutions comparable to microarray analysis (26). With respect to microdeletions, data are available for syndromes such as DiGeorge syndrome, Prader-Willi/Angelman, Cri-du-chat, and del1p36, excluding microdeletions shorter than 3 Mb (27). However, routine testing with digital PCR is not possible as deep sequencing analysis is highly cost-intensive.

### 2.3. Next-generation sequencing NIPT methodology

Next-generation sequencing (NGS) is a massively parallel sequencing technology that is used to determine the order of nucleotides in entire or targeted regions of DNA. It offers ultra-high throughput, scalability, and speed. Unique molecular indexes (UMI) are used to label cell-free DNA after plasma cfDNA is extracted from maternal blood. This process is performed prior to PCR amplification and sequencing to aid in identifying true DNA changes from artifacts introduced during the amplification process. Following library construction, target gene enrichment, and NGS, the data are then analyzed using the distribution of UMIs to predict the variants representative of the cf DNA material used for analysis. The analytical sensitivity for a single nucleotide variant (SNV) is >99%, with test specificity at >99%. Small indels may be detected at a lower sensitivity.

With the developments in NGS, clinical laboratories now provide a plethora of genetic testing for genetic disorders. These include genotyping, single gene analysis, analysis of gene panels, exomes, whole genomes, transcriptome analysis, and analysis of epigenetic changes. This has created novel challenges in the interpretation of the enormous amounts of NGS-generated sequencing data. In this context, the working group of the American College of Medical Genetics (ACMG) along with the Association for Molecular Pathology (AMP) and the College of American Pathologists (CAP) revisited and revised the standards and guidelines for the interpretation of sequence variants. The use of standard terms, which include “pathogenic,” “likely pathogenic,” “uncertain significance,” “likely benign,” and “benign,” is recommended by these guidelines. These terms are used to describe variants identified in Mendelian disorders. The recommendations also describe a procedure for evidence-based classification of the variants into five categories (i.e., population data, computational data, functional data, and segregation data). A second sample of cfDNA is used to confirm pathogenic and likely pathogenic variants using an amplicon-based NGS assay. This method enriches the targeted region using gene-specific primers, followed by deep sequencing methods (>10,000X) to confirm cfDNA variants (28).

## 3. NIPT for chromosomal abnormalities

The focus of early investigations on prenatal diagnosis emphasized the potential role of amniotic fluid cytology in the determination of fetal sex and karyotyping. Chorionic villi sampling procedures have been performed since the 1980s to assess fetal karyotypes, providing an alternative prenatal diagnosis option for the first trimester and paving the way for large-scale utilization of amniocentesis and chorionic villus sampling (CVS) for invasive methods of genetic disorder diagnosis.

Limitations of invasive prenatal testing include the nature of the procedure and the risk of a failed pregnancy. Current literature suggests the invasive procedure-related risk to be <1% (29) but there has been a shift to limit the tests for invasive diagnostic testing, which has accelerated the identification of non-invasive screening tests, especially for cases with a higher risk of fetal aneuploidies. Traditionally, the choice of an invasive testing method for prenatal testing was defined by the advanced maternal age while considering family history and ultrasound findings. However, the use of maternal age only as an index for screening has a very low sensitivity of ~30% and a very high false-positive rate (FPR) of 15% (30). Moreover, advanced maternal age does not represent an increased risk for sex chromosome aneuploidies except for X chromosome non-disjunction errors or triploidy, even with an increased risk for trisomies 21, 13, and 18. Identification of additional biochemical markers of fetal aneuploidies has resulted in the development of two new methods of screening, namely, the triple test, which combines maternal age and serum AFP levels, free β-hCG, and uE3, with a detection rate of ~70%, and the quadruple test, with the addition of inhibin A, with a detection rate of ~75% (30, 31).

The introduction of nuchal translucency (NT) and the combined screening test (CST) in the 1990s revolutionized the field of NIPT. It involves a first trimester CST for T21, T18, and T13 in combination with gestational age, NT, and the multiples of the median of circulating free β-hCG and PAPP-A (32). Research data from Santorum and colleagues (33) reviewed more than 108,000 CSTs and they reported a FPR of 4% and a DR of 90% for trisomy 21, 97% for trisomy T18, and 92% for trisomy 13.

In a nationwide implementation study in the Netherlands, NIPT was conducted on 73,239 pregnant subjects (42% of all pregnancies). Of these, 7,239 pregnant women (4%) chose first-trimester combined testing. Trisomy 21 was detected in 239 women (0.33%). For trisomy 18, 49 pregnancies were positive (0.07%) and for trisomy 13, 55 women were positive (0.08%). Reported rates in this study were comparable to earlier studies, but the positive predictive value (PPV) was higher than expected, reported as 96% for trisomy 21, 98% for trisomy 18, and 53% for trisomy 13 (34).

The demonstration of fetal cell presence in maternal circulation and the presence of cell-free fetal DNA (cffDNA) in maternal plasma and serum, which increases with maternal age, have led to further advancements in the field of NIPT. Fetal heart rate, which is used in some programs during first-trimester screening, has been shown to improve the accuracy of the first-trimester combined test in screening for trisomy 13, but not for trisomy 21 or 18 (33).

The introduction of prenatal screening for aneuploidy has been one of the most successful applications of NIPT (35, 36). Sequencing cffDNA in maternal plasma now enables screening for T21 with a very low FPR. At present, there is a need for the reassurance of healthy progeny from natural conception or from techniques using *in vitro* fertilization (37, 38), which has accelerated the development of NIPT (39).

### 3.1. Abnormalities of the sex chromosome

Varied rates of prevalence for abnormalities in sex chromosomes have been reported. When considered individually, the prevalence rates for SCAs are low, but when combined together, SCAs may have a prevalence rate of ~1% of live births (40). The most prevalent aneuploidies in sex chromosomes include Turner syndrome, Klinefelter syndrome (XXY), XYY syndrome, and XXX. The prevalence rates are approximately 1 in 2,500, 1 in 500 to 1 in 1,000, 1 in 850 to 1 in 3,000, and 1 in 1,000, respectively (41–46). These conditions do not present any symptoms in the immediate period following delivery, except in the case of Turner syndrome. Testing for monosomy X, which is available since 2012, was followed by the introduction of other sex chromosome-linked conditions, including XXX, XXY, and XYY (47). The combined average detection rates (DR) for monosomy X are 89% (83–94%) (47–49), 90% for diplo Y (49), 82% (67–100%) for Klinefelter syndrome (47–49), and 87% (75–100%) for XXX syndromes. In one of the largest studies on sex chromosome abnormalities (SCAs), including a retrospective analysis of more than 67,000 chorionic villus sampling (CVS) karyotypes, the authors report confined placental mosaicism in 23.4% of the cases without ultrasound anomalies, with a PPV of 53% in these cases. Cases with ultrasound anomalies reported a PPV of 98.9% (50). Different research groups have reported varied SCA detection accuracy using the NIPT assay. For example, Deng et al. (51) have reported the PPV for NIPT as 18.39% for monosomy X, 44.4% for trisomy X, 39.29% for 47, XXY, and 75% for 47, XYY. In another study, Zheng et al. (52) have reported a PPV of 44.4% for monosomy X, 58.3% for trisomy X, 100% for 47, XXY, and 50% for 47, XYY. Similarly, in a fetal cfDNA screening test on 9,985 pregnancies, Margotti et al. (53) reported that the estimated PPV for monosomy X was 69.2%, that for trisomy X was 100%, that for 47, XXY was 80%, and that for 47, XYY was 100%. The overall PPV of NIPT in the present study for fetal SCAs was 77.3%. In cases that are NIPT positive for SCAs but have no ultrasound features, diagnostic testing by amniocentesis is generally recommended (50).

Although the screening for SCAs is fairly accurate, it comes with the ethical dilemma of sex selection. Therefore, while the European Society of Human Genetics currently recommends against the use of NIPT for SCAs, the recent practice guideline from the American College of Medical Genetics (ACMG) strongly recommends non-invasive prenatal screening (NIPS) over traditional screening methods for all pregnant patients with single and twin gestations for trisomies 21, 18, and 13. Additionally, it strongly recommends that NIPS be offered to patients to screen for fetal SCAs (54).

### 3.2. Detection of microdeletions/duplications and rare trisomies

Approximately 1.7% of the pregnancies have pathological copy number variations (CNVs) with normal findings. The array of conditions now included for NIPT testing of cell-free DNA (cfDNA) covers DiGeorge, Cri du Chat, Prader Willi/Angelman syndromes, 1p36 deletion, Jacobson syndrome, and Wolf Hirschhorn syndrome. DiGeorge syndrome is the second most common cause of intellectual disability in children, after Down syndrome. The PPV of cfDNA in cases with microdeletions detected remains low, with a dataset from a large sample having a 13% PPV for common microdeletion syndromes such as DiGeorge syndrome, Prader-Willi/Angelman syndrome, Cri-du-chat syndrome, and del1p36 syndrome. However, more recently published data have highlighted the high sensitivity and specificity of the detection of clinically significant CNVs using NIPT. For classic MMSs, PPVs of 75–93% for DiGeorge syndrome (55), 68–80% for 22q11.22 microduplication (55), 50–75% for Prader-Willi/Angelman syndrome (56), and 50% for Cri-du-chat (56) have been reported.

Furthermore, there is no association with other known risk factors, such as maternal age, for trisomies, preventing its clinical application. These syndromes also have a high negative predictive value (NPV). Therefore, although the PPV is low, a negative result could be considered reassuring. Recent clinical experience of retrospective clinical outcomes of patients who received a positive NIPT result tested the scope of NIPT use for the detection of RAAs and large CNVs (>7 Mb) in addition to common aneuploidies. NIPT was successful in the detection of unbalanced reciprocal translocations from carriers of two maternally balanced reciprocal translocations. The detection was highly sensitive for a specific minimal size of one translocated CNV of 15 Mb and an average sequencing depth of ~11 million single-end reads of the corresponding NIPT, both of which are critical parameters for CNV sensitivity (57).

Rare autosomal trisomies (RAT) include placental mosaicism or uniparental disomy, where the fetus inherits both sets of chromosomes from a single parent. In a study published by Pertile et al. (58), only 5 of the 60 cases were true RATs, and the remaining cases were classified as confined placental mosaicism. Except in the case of confined placental mosaicism for T16, which has an established risk to the fetus, the data on the involvement of other chromosomes in confined placental mosaicism remain inconclusive (59).

A large nationwide implementation of NIPT in the Netherlands determined the clinical impact of screening for chromosomal aberrations other than common trisomies on fetal and/or maternal health in 149,318 pregnancies (34). Additional findings other than common aneuploidies were detected in 1 out of every 275 performed genome-wide NIPT and accounted for 35.5% (402/1,132) of all abnormal NIPT results within the TRIDENT-2 cohort. The findings included 196 RATs, 188 structural aberrations, and 18 complex profiles. Follow-up testing using genetic methods indicated an assumed fetal origin in 22.1% of the cases. Assumed placental origin was reported for 52.8% of the cases, and an assumed maternal origin of chromosomal aberrations was estimated for 25.1% of cases. A large variation in PPVs was observed between RATs and SAs (7.7 vs. 44.1%), in line with a previously published study (34). While the study reports a lower PPV for the additional findings, especially for RATs, in comparison to single-common trisomy, the PPV was still higher than the PPV of first-trimester combined testing for trisomy 21, 18, or 13 (combined: 4.4%) (34). There are scarce data addressing the clinical relevance of the additional findings from whole-genome sequencing (WGS)-based NIPT, and it is rare to see the point of view from the patient's perspective. Clinical data remain scarce, and large-scale clinical validation is still required before professional societies recommend its clinical use. It is often difficult to assemble a patient cohort closely resembling a clinical population, making the validation process difficult and time-consuming. Furthermore, conditions that are extremely rare in a population have less rational basis to be included in NIPT screening as their prevalence rates remain unknown.

### 3.3. Detection of triploidies through NIPT

Very thin placentas and very low cffDNA are generally seen in triploidies, making them extremely difficult to be detected through NIPT, even when features are seen in ultrasound scans, along with the presence of abnormal biomarkers suggestive of triploidy in up to 90% of cases (60). An extra haplotype is sometimes encountered in single nucleotide polymorphism (SNP)-based non-invasive prenatal testing (NIPT). This may be attributed to either an undetected twin or triploidy. In an analysis of 515,804 women receiving SNP-based NIPT, 1,005 were positive for an extra haplotype (1 in 513). Outcomes of pregnancy were available for 773 cases. Notably, 11% of cases had confirmed or suspected triploidy, 65% of those were attributed to a vanished twin, and 10% of the available outcomes reported a loss of pregnancy. Ultrasound is recommended to establish viability, evaluate for viable or vanished twins, and detect findings consistent with triploidy in cases with an extra haplotype (61).

### 3.4. Genome-wide NIPT for the detection of monogenic diseases

The use of NIPT for the detection of monogenic diseases was first reported in the year 2000 and has since been followed by many studies establishing the proof of principle in the detection of several single-gene diseases by analyzing cffDNA early in pregnancy (62). There are a small number of commercially available non-invasive prenatal tests that can detect monogenic diseases; however, the tests are designed to either detect autosomal recessive conditions or autosomal dominant and *de novo* conditions. The first monogenic disease detected through NIPT was achondroplasia, and later NIPT was introduced into clinical practice. Later, other autosomal dominant monogenic diseases were also included. These are Crouzon syndrome, thanatophoric dysplasia, osteogenesis imperfecta, Apert syndrome, torsion dystonia, and several others (63). Mohan et al. (64) published initial clinical experience with NIPT-SGD, focusing on a set of 30 genes, to search for pathogenic or likely pathogenic variants that were known to be involved in 25 dominant conditions. The conditions included Noonan spectrum disorders, skeletal disorders, craniosynostosis syndromes, Cornelia de Lange syndrome, Alagille syndrome, tuberous sclerosis, epileptic encephalopathy, *SYNGAP1*-related intellectual disability, CHARGE syndrome, Sotos syndrome, and Rett syndrome. CffDNA isolated from maternal plasma was used for the analysis. In a cohort enriched for pregnancies at increased risk for these disorders, 5.7% (125/2,208) tested positive. In addition to identifying causal gene variants in fetuses with abnormalities, the test detected previously unidentified carrier parents. Analysis of cases with confirmatory follow-up testing revealed no false-positive or false-negative results. The study reports an observed test-positive rate of 0.4% (6/1,562) for cases without ultrasound abnormalities or a family history. The results suggest the benefits of using NIPT for pregnancies with apparently normal ultrasound results. They went on to suggest that NIPT-SGD could be offered in the first trimester (in conjunction with NIPT to screen for aneuploidy) when fetal anatomical abnormalities are typically not visible in the conditions analyzed in their research. In this study, 99 cases had abnormal sonographies. Of these, 30 cases (30.3%) were identified only after the ultrasound abnormalities were detected in the third trimester, which is beyond the recommended time window for invasive testing via CVS or amniocentesis.

Carrier screening with reflex single-gene non-invasive prenatal screening has been developed to screen for a small number of autosomal recessive conditions such as cystic fibrosis, hemoglobinopathies, and spinal muscular atrophy via next-generation sequencing. It is possible to identify a paternally inherited fetal variant that is absent in the maternal genome, as seen in *CFTR* screening for cystic fibrosis, or identify variants in the fetus absent in the maternal genome by investigating polymorphic regions using parental haplotypes as a reference (65). The implementation of this testing is beneficial in cases where pregnancy is unplanned, where gestational prenatal carrier testing is late, where the partner is unavailable for testing, and/or to expand the accessibility of testing for qualified, uninsured patients.

The detection of monogenic conditions is not without problems, particularly in the case of maternally inherited alleles. This is because the inherited allele is genetically identical to the maternal allele, lowering its fetal detection. While NIPT for paternally inherited monogenic conditions is already in clinical practice, it is not yet applicable to maternally inherited conditions. Further limitations of testing for the detection of monogenic conditions include a relatively small cohort of positive cases compared to most individuals who receive negative results, and additional studies with larger cohorts are necessary to identify the full clinical impact of NIPT for monogenic diseases.

Recently, non-invasive prenatal multi-gene sequencing was developed and is commercially available to screen for monogenic diseases. It was designed to detect *de novo* and paternally inherited pathogenic and likely pathogenic variants in circulating cffDNA present in maternal blood. The test can be performed as early as 9 weeks for singleton pregnancies. The panel includes 30 genes that were selected based on single-gene etiology, high *de novo* incidence-causing disease, and were either inherited in an autosomal dominant or X-linked manner. Validation for this test was completed in two phases and showed the ability to detect benign and disease-causing DNA variants using spike-in samples (DNA with known variants paired with known maternal and paternal samples) and samples from pregnant women. Phase one included a study design to determine the accuracy, sensitivity, and specificity of the assay and was based on the detection of fetal variants by comparison of the sequencing results of plasma DNA and genomic DNA of parental samples (from both patient and spike-in samples). Fetal fraction was validated using highly polymorphic SNPs and SRY by examining two types of informative loci to estimate fetal fraction from cell-free DNA.

Phase two assay validation removed the paternal sample, only requiring the maternal sample to identify variants in the fetus using plasma cell-free DNA and maternal DNA to inform the final report. A statistical model was developed to evaluate the variants in question using unique molecular indexes (UMIs) to label individual DNA molecules in question to assess if changes are truly derived from fetal DNA.

In the initial phase of validation, the fetal fraction was calculated based on SNP information from maternal, paternal, and egg donor samples, whereas phase two allowed for fetal fraction calculation based on the maternal sample only without paternal data or egg donor data using a regression algorithm. For variant interpretation, NGS results from maternal cfDNA and maternal genomic DNA were used for the interpretation of the variants and calculation of the fetal fraction (66). Clinical performance from both validation studies has been previously reported, as both analytical sensitivity and specificity are >99% (66). While results from monogenic NIPT are highly accurate, limitations exist due to the limited data and validation studies performed. Screening for monogenic diseases does not replace diagnostic testing for pregnancies with abnormal clinical findings, nor can the testing methodology detect exonic, gene, or chromosomal copy number changes.

## 4. NIPT in the management of multifetal pregnancies

There is an increased risk of a broad range of pregnancy complications and adverse outcomes with multifetal pregnancies. While only a minority of twin pregnancies may be monochorionic, they are responsible for higher perinatal morbidity and mortality. Chorionicity is a risk factor in pregnancies and is responsible for poor outcomes in twin pregnancies. The risk of twin-to-twin transfusion syndrome, twin anemia-polycythemia syndrome, and twin reversed arterial perfusion is high in monochorionic twins. Chorionicity can be established during routine first-trimester ultrasound scans in patients who have access to early diagnostic ultrasound (67).

Current research data validate that there is indirect evidence to conclude that the use of cf-DNA testing has resulted in improvements in fetal trisomy screening in cases of twin pregnancies. Moreover, literature data also point to the direct benefit of using cfDNA-based screening for common trisomies in twin pregnancies. This is especially true for trisomy 21, where cfDNA testing provides higher positive predictive values among twin pregnancies compared with traditional serum and NT-based screening for twin pregnancies (24, 68). In cases where chorionicity assignment using ultrasound is uncertain or there is late detection of twin pregnancies, NIPT can be used to evaluate zygosity. Monozygotic pregnancies do not always imply monochorionicity, but dizygotic twins are highly likely to be dichorionic. One of the challenges in NIPT is the presence of two or more fetal genomes in cf-DNA, each present in different concentrations. This may result in higher rates of uninterpretable tests. In certain cases, a vanished twin identified via cf-DNA testing may be helpful in obstetric management and patient care. NIPT is expected to play a pivotal role in the clinical management of women with multiple pregnancies.

In multiple gestational pregnancies, cfDNA testing is offered as complementary to first-trimester ultrasound screening. In addition to determining chorionicity in twin pregnancies, first-trimester ultrasound scans identify maternal pathology and abnormalities in the developing fetus (e.g., increased NT and major structural congenital abnormalities such as anencephaly), which may affect the outcomes of multifetal gestations. As discussed, NIPT provides high-quality screening for aneuploidy and information about embryo zygosity. Taken together, ultrasound combined with NIPT facilitates early diagnosis of serious adverse conditions and allows clinicians to make informed decisions on the continuation or termination of pregnancy. Furthermore, the application of these technologies in twin pregnancies illustrates the synergy between imaging and laboratory diagnostic methods.

## 5. Limitations of NIPT using the cffDNA test in clinical practice and ethical concerns

The circulating cffDNA in maternal blood has its origins in the placenta. Therefore, there is a limitation that the procedure may generate false positives during NIPT, as the detected abnormalities may be restricted to the placenta in cases of confined placental mosaicism without any effect on the fetus. Additionally, in cases where chromosomal abnormalities have a maternal origin, which include those having their origin in a maternal tumor, a low fetal DNA fraction may result in inconclusive, false-positive, or false-negative results due to inefficient sequencing depth or low yields of fetal DNA templates.

Current research indicates additional uses of NIPT in the detection of other trisomies, abnormalities in sex-chromosomes, and anomalies at a sub-chromosomal level associated with rare diseases, as well as the unmet need to prioritize the development of gene panels that allow for the comprehensive diagnosis of severe childhood-onset disorders.

In comparison to other screening methodologies, the costs associated with NIPT remain high and are more or less similar to invasive tests with karyotyping. The second limitation includes the rates of failed analyses, which pose a significant challenge in case management as they often require confirmation via invasive methods such as amniocentesis.

NIPT has experienced rapid diffusion, and it carries the potential to disrupt traditional prenatal testing pathways. NIPT is not a diagnostic test, and clinical practice guidelines recommend that test results that are positive using NIPT must be confirmed using invasive fetal testing methods such as amniocentesis. However, the introduction of NIPT has been associated with a decreased uptake of diagnostic testing. Furthermore, since NIPT was introduced, the number of invasive diagnostic procedures performed has shown a decline (69). Apart from the costs associated with additional tests being included in NIPT, the expansion raises ethical as well as policy-based questions on whether NIPT should be expanded to include tests where rates of prevalence in a population are unknown. It also raises the question of clinical utility and concerns related to an informed choice. Decades of research and medical literature on prenatal testing emphasize the challenge for clinicians to provide unbiased information to patients in a way that facilitates informed choice, and the rapidly evolving nature of NIPT adds to this challenge. To this end, the ACOG clearly states that physicians should be aware of the potential of NIPT to generate false-positive and false-negative results and that it is “not equivalent to diagnostic testing”. As per ACOG, patients with a positive screening test result for fetal aneuploidy should undergo genetic counseling and a comprehensive ultrasound evaluation, with an opportunity for diagnostic testing to confirm results. Additionally, clinicians have described several limitations hindering the expansion of NIPT. Most importantly, lack of knowledge about the latest advancements in the field and their own comfort level with the use of NIPT have been cited as important limitations. These have been attributed to the lack of clinical evidence as well as a lack of education and guidelines (69).

## 6. Conclusion

There is no doubt that NIPT has revolutionized the field of prenatal testing due to its improved analytical performance over other screening methods and its non-invasive nature over traditional prenatal diagnostic techniques. The technical advancements in detection methods for a condition may not correspond to the clinical benefit to a population. Therefore, the benefits and risks associated with screening programs must be carefully considered before implementation. The guidelines and criteria laid down by the World Health Organization on the use of cfDNA screening should be taken into consideration when implementing expanded test availability for cell-free DNA analysis. Conditions such as microdeletions and duplications are extremely rare in a population, and their prevalence has not been defined. Furthermore, their consequences in the prenatal period cannot be reliably predicted due to a lack of data and scientific evidence.

The scope of NIPT should be responsibly expanded, and informed choice must be taken as a precondition. When NIPT includes an expanded set of tests for chromosomal or sub-microscopic abnormalities, it must be accompanied by an improvement in pre-test counseling for ethical reasons. The expansion of the NIPT panel of tests should be clinically justified. Finally, healthcare resources invested in the reimbursement of NIPT should be precisely distributed based on scientific evidence, benefit, and utility.

## 7. What is already known about this topic?

The detection of cell-free fetal DNA (cffDNA) in maternal plasma was first reported in 1997. This discovery paved the way for the development of a new field in prenatal diagnosis. Based on the discovery, non-invasive prenatal testing (NIPT) is now being used by millions of pregnant women annually.

## 8. What does this study add?

This review highlights the important genetic conditions where basic scientific discoveries can be translated and applied in the clinic for improved diagnosis.

It also touches upon the ethical questions about the implications of the expansion of this technology where the disease conditions or its impact on the developing fetus are not completely understood.

## Author contributions

MA: Conceptualization, Funding acquisition, Writing—original draft, Writing—review & editing. SB: Writing—review & editing. RF: Writing—review & editing. AM: Writing—review & editing. NA: Writing—original draft. EF: Writing—review & editing.
